# Dr. Robert J. Menzie

**Published:** 1915-06

**Authors:** 


**Dr. Robert J. Menzie,** Buffalo 1866, died in Caledonia, April 20, aged 82, of cerebral haemorrhage.

